# Unjustified Restrictions on Letters to the Editor

**DOI:** 10.1371/journal.pmed.0020126

**Published:** 2005-05-31

**Authors:** Douglas G Altman

**Affiliations:** Cancer Research UK/NHS Centre for Statistics in MedicineOxfordUnited Kingdom

Editors of medical journals accept that published research should be open to comment and correction in published correspondence ([1]; Box 1). “Post-publication peer review” enables comments on, clarifications of, and corrections to published research. All journals should have a correspondence page for this purpose.

**Box 1. “Editors Should Promote Self-Correction in Science and Participate in Efforts to Improve the Practice of Scientific Investigation by:**

- “Publishing corrections, retractions, and letters critical of articles published in their own journal.
- “Playing an active role in investigating and preventing fraud.
- “Taking responsibility for improving the level of scientific investigation and medical writing in the larger community of potential authors.
- “Giving authors an opportunity to review and approve edited manuscripts before they are published.
- “Participating in efforts to detect and prevent publication bias—for example, by collaborating with registries of controlled trials and publishing protocols.”

Source: [1].

I previously criticised the effective “statute of limitations” in several leading general medical journals “whereby authors of papers are immune to disclosure of methodological weaknesses once some arbitrary (short) period has elapsed” [2]. Such a time limit discourages post-publication peer review, with potential correspondents deterred by the short and unambiguous deadline. I suggested that journals with such a policy should reconsider. The word limit on that article precluded additional adverse comments on journals' word limits for letters, although they were presented in Table 2 of that article [2].

Subsequently, three of the six journals did revise their instructions [3–5], but each imposed tougher restrictions on letters, reducing either the maximum time limit, the maximum length, or both. The strictest current requirements are a two-week limit by *The Lancet* and a 175-word limit by the *New England Journal of Medicine*.

Editors are seemingly falling over themselves to speed up and shorten letters, but this behaviour is inappropriate for a scientific journal. The key characteristic of science is not its infallibility, a quality it clearly does not and cannot have, but its self-correcting ability. The decision by medical editors to stifle debate is misguided [2,6]. A time limit, especially a very short one, signals that speed is more important than content, that convenience takes precedence over science. While it is reasonable to encourage early comments, there should be no time limit on comments aimed at clarifying or criticising study methodology. Likewise, it will often be impossible to explain the subtleties of methodological problems in 400 words, and impossible in only 175. Additional restrictions on the number of authors and references are also questionable.

I am disappointed that *PLoS Medicine* has imposed a time limit of four weeks on correspondence. As explained above, I believe that such a limit is mistaken. The word limit of 750 words is generous by comparison to established general medical journals, but even this should be open to flexibility should the circumstances merit it.

In this world of Web-based journals and Web pages for print journals there is no real cost to permitting longer and later letters on the web while keeping the print version timely and terse.

## References

[pmed-0020126-b1] World Association of Medical Editors (2001). Report of the World Association of Medical Editors (WAME): An agenda for the future. http://www.wame.org/bellagioreport_1.htm.

[pmed-0020126-b2] Altman DG (2002). Poor-quality medical research: What can journals do?. JAMA.

[pmed-0020126-b3] Mullan Z (2003). Lancet correspondence: Old letters, new rules. Lancet.

[pmed-0020126-b4] Curfman GD, Graham A, Lindenfelser L, Anderson KR, Drazen JM (2003). Innovations in correspondence. N Engl J Med.

[pmed-0020126-b5] Davies S (2003). New edicts for letters to the editor. BMJ.

[pmed-0020126-b6] Metcalfe S (2003). Old letters, new rules. Lancet.

